# Melatonin attenuates smoking-induced atherosclerosis by activating the Nrf2 pathway via NLRP3 inflammasomes in endothelial cells

**DOI:** 10.18632/aging.202829

**Published:** 2021-04-04

**Authors:** Zhewei Zhao, Xuebin Wang, Rui Zhang, Baitao Ma, Shuai Niu, Xiao Di, Leng Ni, Changwei Liu

**Affiliations:** 1 Department of Vascular Surgery, Peking Union Medical College Hospital, Peking Union Medical College and Chinese Academy of Medical Sciences, Beijing, China

**Keywords:** melatonin, cigarette smoking, pyroptosis, Nrf2 pathway, atherosclerosis

## Abstract

Substantial evidence suggests that the effects of smoking in atherosclerosis are associated with inflammation mediated by endothelial cells. However, the mechanisms and potential drug therapies for smoking-induced atherosclerosis remain to be clarified. Considering that melatonin exerts beneficial effects in cardiovascular diseases, we examined its effects on cigarette smoke-induced vascular injury. We found that cigarette smoke extract (CSE) treatment induced NLRP3-related pyroptosis in human aortic endothelial cells (HAECs). CSE also induced ROS generation and upregulated the Nrf2 pathway in HAECs. Furthermore, pretreatment of HAECs with Nrf2-specific siRNA and an Nrf2 activator revealed that Nrf2 can inhibit CSE-induced ROS/NLRP3 activation. Nrf2 also improved cell viability and the expression of VEGF and eNOS in CSE-treated HAECs. In balloon-induced carotid artery injury model rats exposed to cigarette smoke, melatonin treatment reduced intimal hyperplasia in the carotid artery. Mechanistic studies revealed that compared with the control group, Nrf2 activation was increased in the melatonin group, whereas ROS levels and the NLRP3 inflammasome pathway were inhibited. These results reveal that melatonin might effectively protect against smoking-induced vascular injury and atherosclerosis through the Nrf2/ROS/NLRP3 signaling pathway. Overall, these observations provide compelling evidence for the clinical use of melatonin to reduce smoking-related inflammatory vascular injury and atherosclerosis.

## INTRODUCTION

Cigarette smoking has long been a major public health challenge owing to the high rates of associated morbidity and mortality. Smoking was estimated to be the cause of roughly one in five deaths among adult Chinese male from 2010-2019 [1] and has been strongly associated with atherosclerosis and cardiovascular diseases (CVD) [2]. Substantial evidence suggests that the effects of smoking in atherosclerosis is linked to oxidative stress and inflammation, which cause the morphological and functional dysfunction of the vascular endothelium [3]. However, the exact mechanistic basis remains to be established.

Atherosclerosis is characterized by chronic progressive endothelial injury and inflammation-induced vascular intimal lesions [4]. Research in this area has shown that pyroptosis is a critical player in the pathogenesis of atherosclerosis [5]. Pyroptosis is distinct from apoptotic cell death, instead representing a form of highly inflammatory necrotic cell death wherein the plasma membrane ruptures and inflammatory factors such as interleukin (IL)-1β and IL-18 are release along with other components from the cytoplasm [6].

Smoking can cause both inflammation and oxidative stress, thereby damaging the function of the endothelium [7]. However, there has been little discussion about the relationship between smoking-induced oxidative stress and pyroptosis. A recent study has revealed that nicotine induces endothelial cell pyroptosis through the reactive oxygen species (ROS)/nucleotide-binding oligomerization domain-like receptors pyrin domain containing 3 (NLRP3) axis [8], indicating that the ROS pathway may interact with the pyroptosis-related pathway in inflammation activation.

Nuclear Factor Erythroid 2-Related Factor 2 (Nrf2) is considered the key transcription factor responsible for controlling the activity of key antioxidant factors including heme oxygenase-1 (HO-1) [9]. Nrf2 pathway activation occurs under stressful conditions and is essential for sensing oxidative stress and in protecting cells against ROS [10]. Nrf2 has been reported to play protective roles in myocardial ischemia and reperfusion injury [11], sepsis [12], and neurodegenerative diseases [13]. Furthermore, Nrf2 has been shown to attenuate inflammation in smoking-induced chronic obstructive pulmonary disease/emphysema and asthma [14, 15]. However, the mechanisms underlying Nrf2 involvement in smoking-induced vascular endothelial injury remain largely unknown.

Melatonin, mainly secreted by the pineal gland, has been indicated as the main chronobiotic hormone, because of the diurnal rhythm of the changes to its concentration in the blood [16]. Since first reported, the functions of melatonin as a highly effective antioxidant have been repeatedly confirmed [17]. Melatonin is also considered to have low side effects as no major adverse events have been reported in its clinical trials [18]. In our previous study, melatonin was found to attenuate cigarette smoking-associated inflammation and oxidative damage [3, 19]. We thus hypothesized that melatonin would also be beneficial in the context of smoking-induced pyroptosis.

This study analyzed the impact of melatonin in cigarette smoke-induced endothelial damage via inhibition of pyroptosis in endothelial cells through the Nrf2 pathway.

## RESULTS

### *In vitro* analysis

### Treatment with cigarette smoke extract induces human aortic endothelial cell pyroptosis

**Figure 1:**
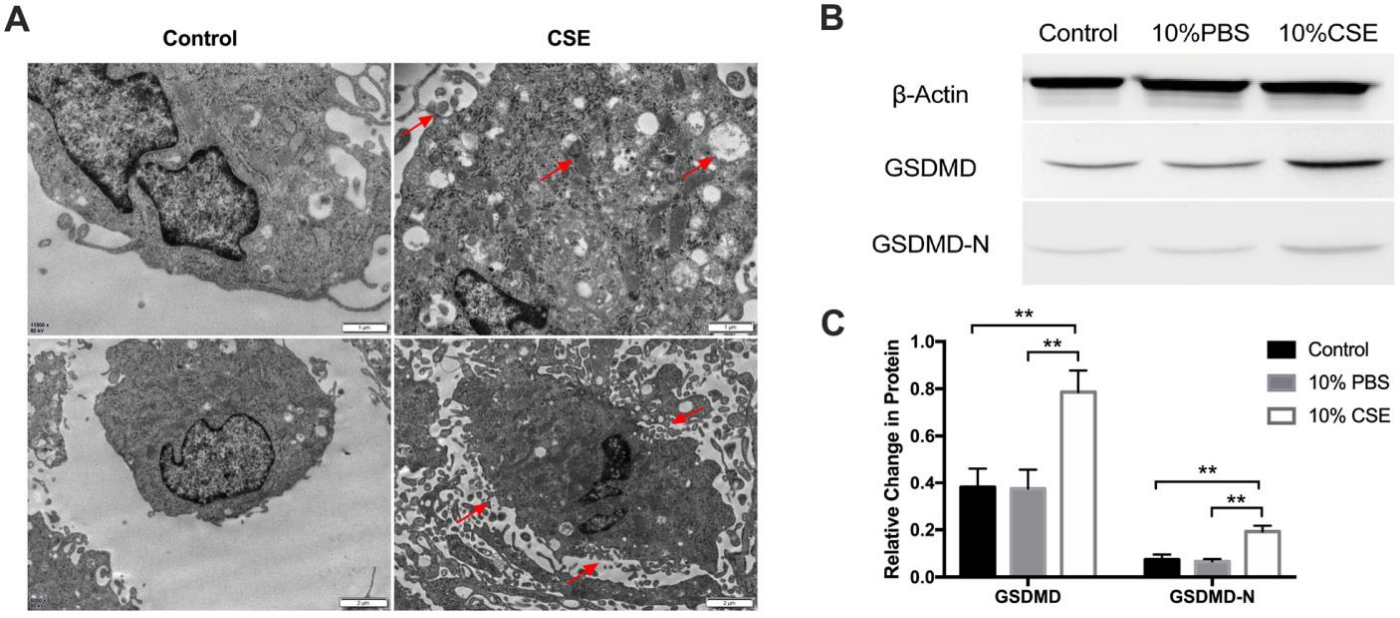
**CSE treatment induced pyroptosis in HAECs.** (**A**) Morphology of HAECs treated with and without CSE imaged using electron microscopy. Autophagosomes, cytoplasmic outflow and cell membrane break indicated by red arrows. (**B**, **C**) The protein levels of GSDMD and GSDMD-N were upregulated in HAECs after treatment with CSE, as indicated by western blot results. β-Actin was used as an internal control. **p < 0.01. The data are represented as mean ± SD (*n* = 3).

Our previous studies have demonstrated that cigarette smoke extract (CSE) exposure in a rat model system aggravated lumenal stenosis and induced inflammatory cytokine expression following balloon-induced carotid artery damage [19]. To further investigate the effect of CSE on human aortic endothelial cells (HAECs), we analyzed the classical pyroptosis-related pathway. We began by assessing the changes in cell morphology following CSE treatment. Morphological changes after CSE treatment were determined by transmission electron microscopy. As shown in Figure 1A, HAECs showed more autophagosomes, cytoplasmic outflow, cell membrane breakdown, and organelle edema. We then examined the protein expression level of Gasdermin D (GSDMD), the key executioner of the pyroptotic pathway, and the N-terminal GSDMD cleavage product (GSDMD-N), which is capable of inducing pyroptosis. Western blotting results showed that GSDMD and GSDMD-N were significantly rose following exposure to CSE relative to that in the control and PBS groups (Figure 1B, 1C). These results suggested that CSE treatment could induce pyroptosis in HAECs.

### The NLRP3 inflammasome pathway plays a role in CSE-induced pyroptosis in HAECs

**Figure 2:**
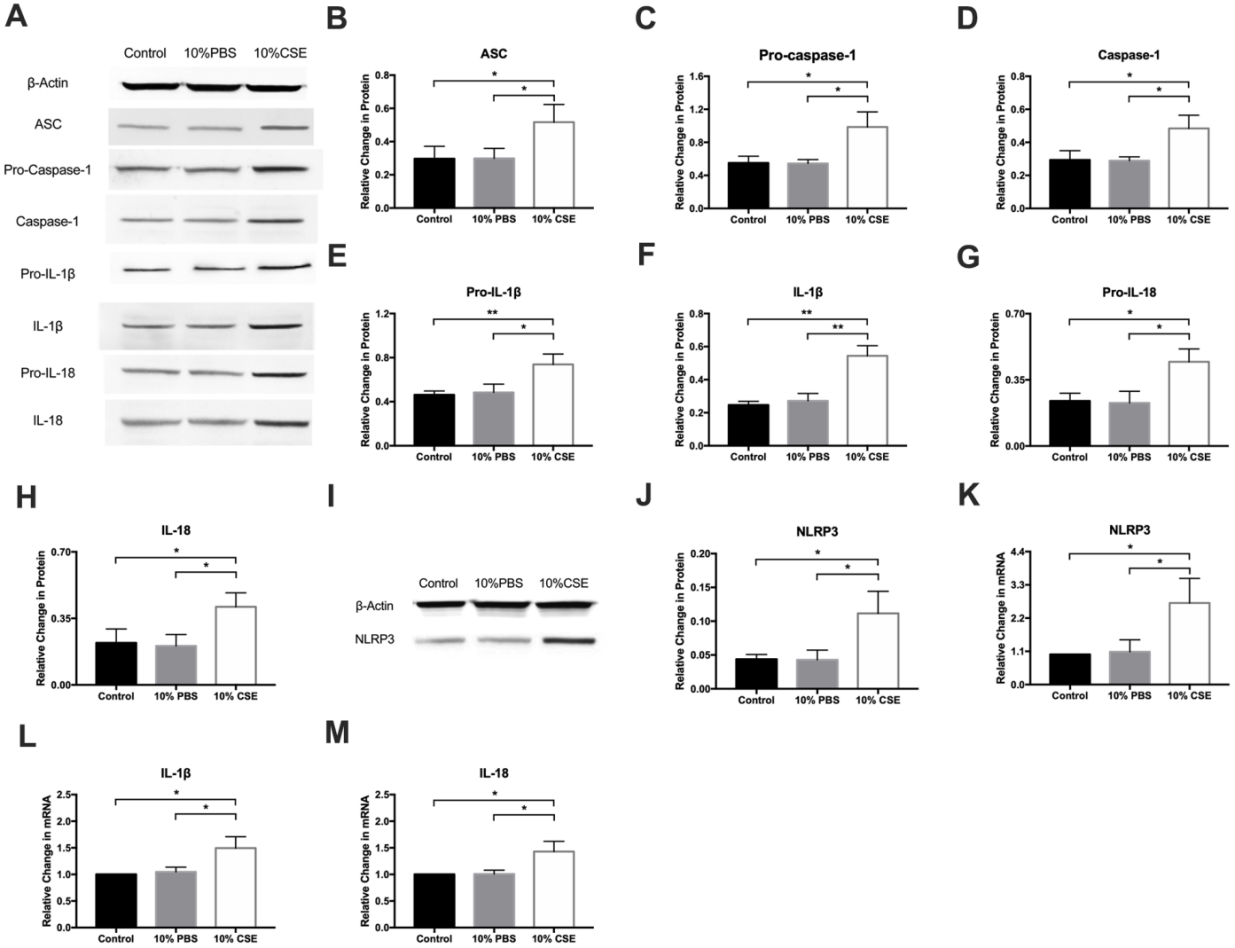
**NLRP3 inflammasome pathway is involved in CSE-induced pyroptosis in HAECs.** (**A**–**H**) The protein levels of ASC, Pro-Caspase-1, Caspase-1, Pro-IL-1β, IL-1β, Pro-IL-18 and IL-18 were upregulated in HAECs after treatment with CSE, as indicated by western blot results. (**I**, **J**) The protein levels of NLRP3 was increased in HAECs after treatment with CSE, as indicated by western blot results. β-Actin was used as an internal control. (**K**–**M**) The mRNA level of NLRP3, IL-1β, and IL-18 was increased in HAECs after treatment with CSE. *p < 0.05, **p < 0.01. The data are represented as mean ± SD (*n* = 3).

Pyroptotic cell death is associated with key morphological changes including the swelling of cells, osmotic lysis, and plasma membrane rupture [20]. Pyroptosis activation requires ASC dependent caspase-1, a key protein in the cleavage and secretion of active IL-1β and IL-18. We measured the expression of pyroptosis-associated molecules, including ASC, pro-caspase-1, caspase-1, pro-IL-1β, IL-1β, pro-IL-18, and IL-18 via western blotting. After CSE treatment, levels of all of these proteins rose significantly (Figure 2A–2H). Of several inflammasome complexes known to activate caspase-1, the most commonly evaluated is the NLRP3 inflammasome, which is a multiprotein platform that is activated upon cellular stress or infection. Western blotting results revealed that NLRP3 expression was remarkably elevated in the CSE group (Figure 2I, 2J). Real-time quantitative PCR assay results showed that CSE stimulation markedly enhanced NLRP3, IL-1β, and IL-18 expression relative to control (Figure 2K–2M). These experiments provide strong evidence for the NLRP3 inflammasome pathway as a mediator of CSE-induced pyroptosis in HAECs.

### CSE induces ROS generation and upregulates the Nrf2 pathway in HAECs

**Figure 3:**
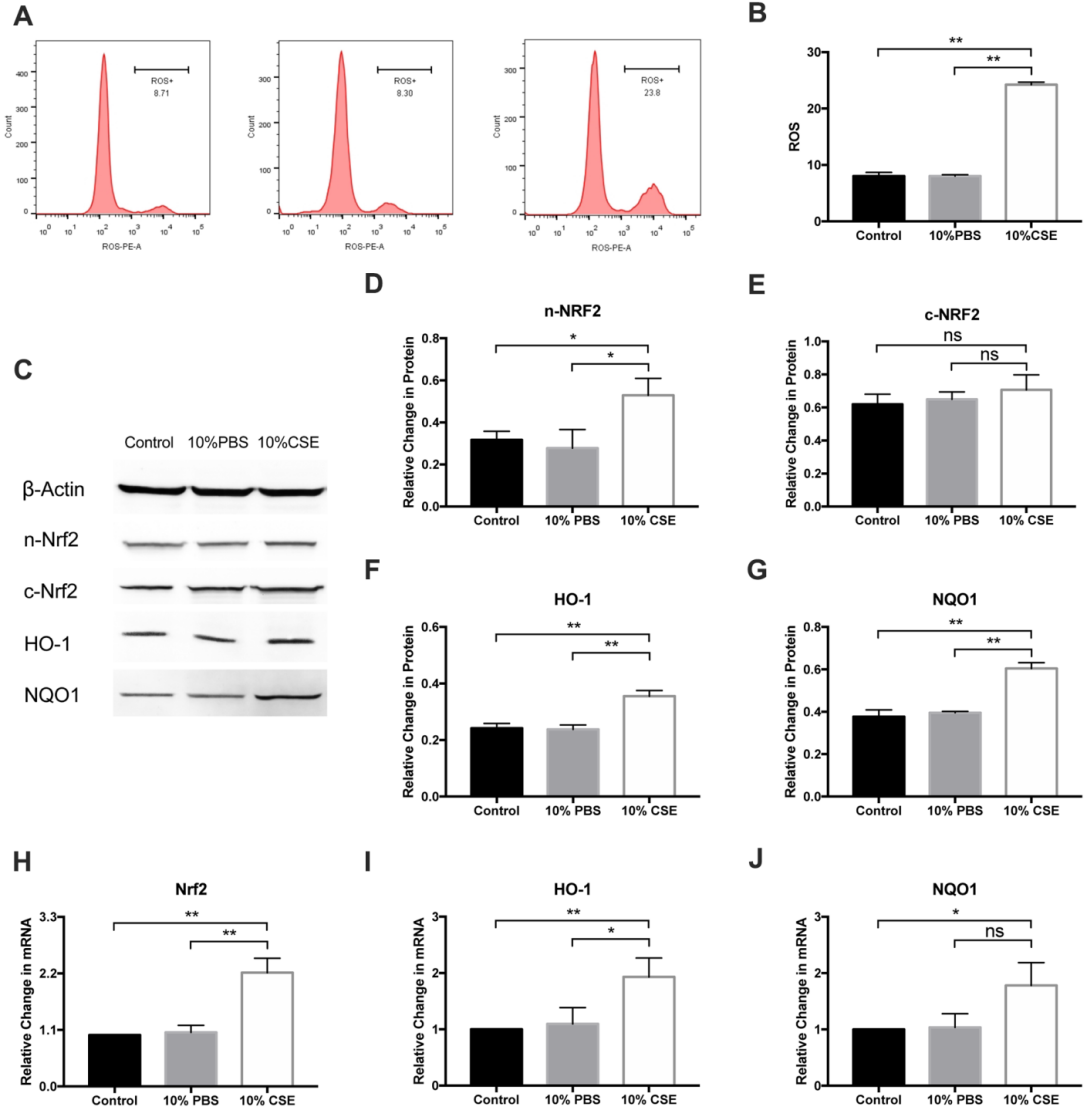
**CSE induced ROS generation and upregulated NRF2 pathway in HAECs.** (**A**, **B**) Cellular ROS level was measured with flow cytometry, and ROS level was promoted after CSE treatment. (**C**–**G**) Western blotting on protein level of n-Nrf2 and c-Nrf2 was performed to analyze Nrf2 nuclear translocation, suggesting that CSE increased Nrf2 nuclear translocation. The protein levels of HO-1 and NQO1 were upregulated in HAECs after treatment with CSE, as indicated by western blot results. β-Actin was used as an internal control. (**H**–**J**) The mRNA levels of Nrf2, HO-1 and NQO1 were upregulated in HAECs after treatment with CSE, as indicated by RT-PCR results. *p < 0.05, **p < 0.01, *ns*, not significant. The data are represented as mean ± SD (*n* = 3).

ROS and oxidative stress are known to be correlated with pyroptosis. ROS is likely to participate in NLRP3 activation, thereby enhancing the inflammatory response [21]. To evaluate how CSE impacts HAEC oxidative stress, we employed dihydroethidium (DHE)-based ROS detection using flow cytometry. As shown in Figure 2A, CSE pretreatment could increase H_2_O_2_-induced ROS rapidly (Figure 3A, 3B). Nrf2 is well-known to induce the expression of ROS-detoxifying enzymes, which prevent ROS accumulation. A previous study has shown that the Nrf2 pathway is protectively upregulated by CSE-induced ROS [22]. In this study, cytosolic and nuclear proteins were extracted from CSE-treated HAECs and analyzed for Nrf2 expression via western blotting. CSE increased the Nrf2 nuclear translocation (Figure 3C–3E). Moreover, levels of HO-1 and NQO1, which are Nrf2 targets, were also enhanced after CSE treatment (Figure 3F, 3G). Further, Nrf2, HO-1, and NQO1 mRNA levels were markedly elevated in the CSE group compared relative to the control (Figure 3H–3J), suggesting that CSE treatment upregulates the NRF2 pathway in HAECs.

### Nrf2 negatively regulates CSE-induced NLRP3 inflammasome activation

**Figure 4:**
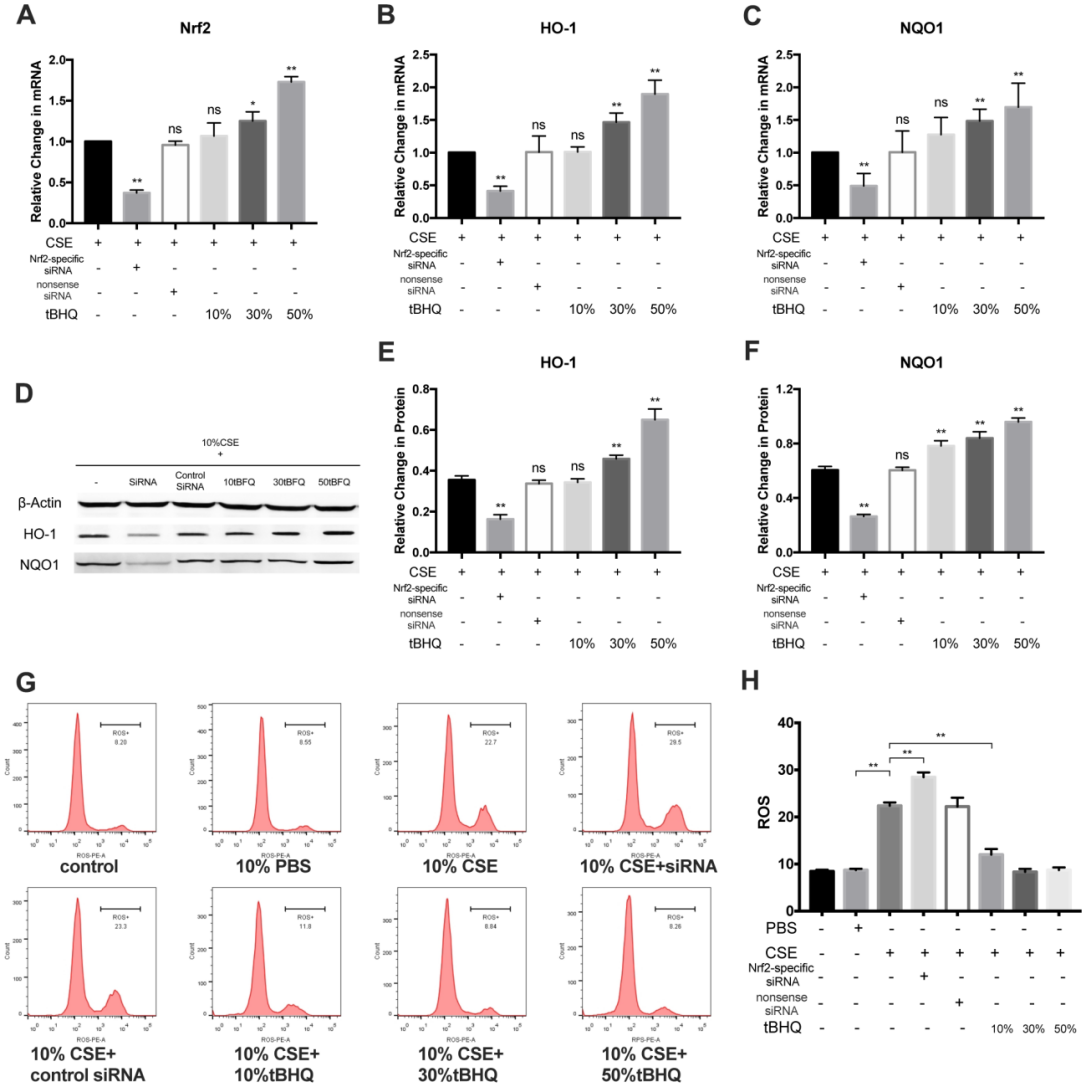
**Nrf2 negatively regulated CSE-induced ROS level.** (**A**–**F**) RT-PCR on mRNA level of Nrf2, HO-1 and NQO1 and western blotting on protein level of HO-1 and NQO1 was performed. (**G**, **H**) Cellular ROS level was measured with flow cytometry, and CSE-induced ROS level was negatively regulated by Nrf2 pathway. *p < 0.05, **p < 0.01, *ns*, not significant, compared with CSE group. The data are represented as mean ± SD (*n* = 3).

**Figure 5:**
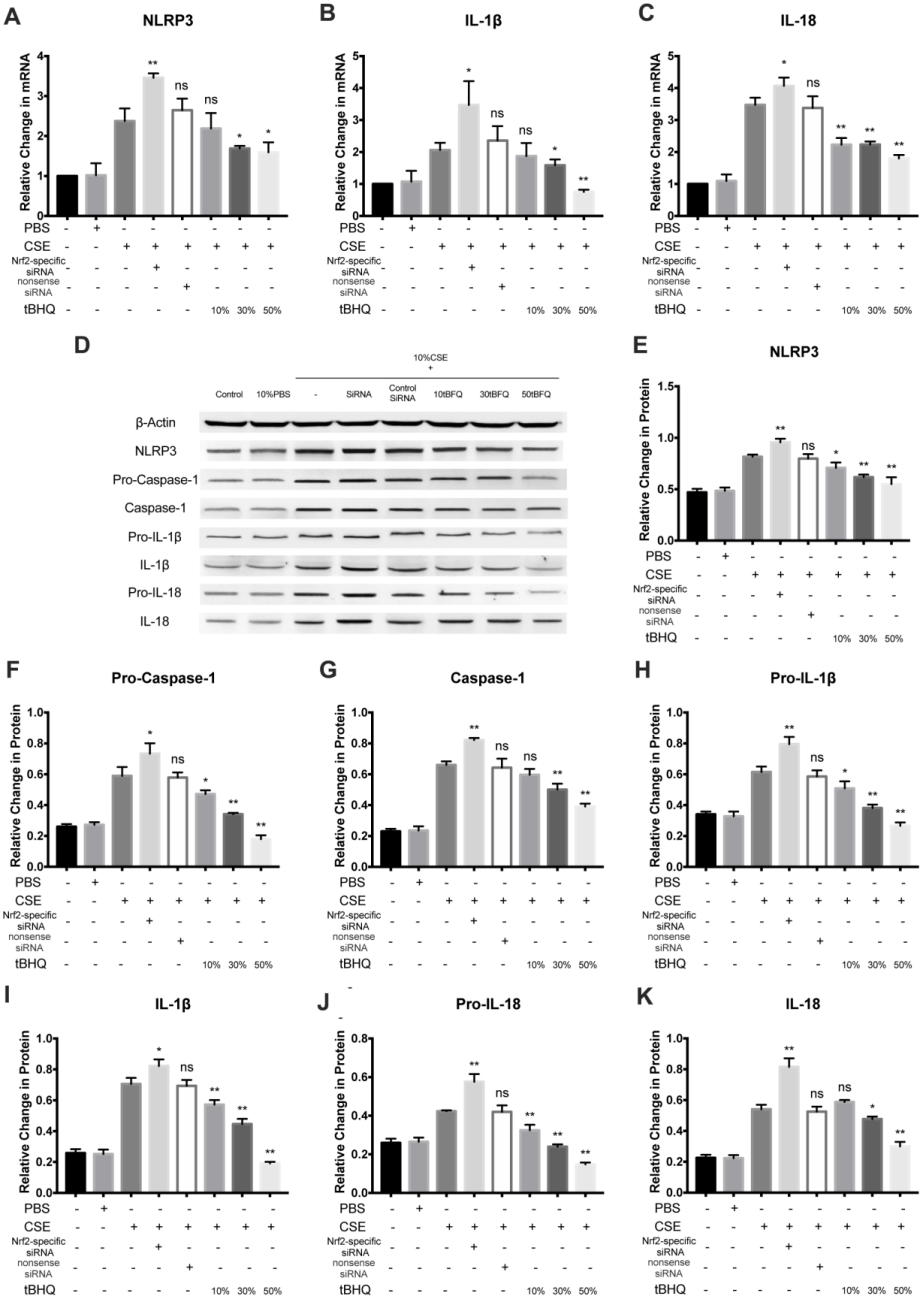
**Nrf2 negatively regulated CSE-induced NLRP3 inflammasome activation.** (**A**–**C**) The mRNA level of NLRP3, IL-1β and IL-18 was detected by RT-PCR. (**D**–**K**) NLRP3, Pro-Caspase-1, Caspase-1, Pro-IL-1β, IL-1β, Pro-IL-18 and IL-18 were detected by Western blot. *p < 0.05, **p < 0.01, *ns*, not significant, compared with CSE group. The data are represented as mean ± SD (*n* = 3).

To explore how Nrf2 regulates the activation of the NLRP3 inflammasome, HAECs were pretreated with Nrf2-specific siRNA and the Nrf2 activator tBHQ before CSE treatment. The Nrf2, HO-1, and NQO1 mRNA levels were significantly inhibited in the Nrf2-specific siRNA group whereas they were stimulated in the tBHQ groups (Figure 4A–4C). The HO-1 and NQO1 protein levels were also remarkably downregulated by Nrf2 siRNA and upregulated by tBHQ treatment (Figure 4D–4F). In addition, the activation effect increased with increasing tBHQ concentration. We then detected the total ROS levels in each group by DHE staining. Fluorescence imaging indicated that pretreatment with Nrf2 siRNA could increase the ROS level, whereas pretreatment with tBHQ could reduce the ROS level. (Figures 4G, 4H). Further, the mRNA expression of NLRP3, IL-1β, and IL-18 were also markedly increased in the Nrf2-specific siRNA group and decreased in the tBHQ groups (Figure 5A–5C). NLRP3, pro-caspase-1, caspase-1, pro-IL-1β, IL-1β, pro-IL-18, IL-18, IL-1β, and IL-18 protein levels increased in the Nrf2-specific siRNA group and decreased in the tBHQ groups (Figure 5D–5K). These data show that the ROS levels and NLRP3 inflammasome are negatively regulated by the Nrf2 pathway.

### Nrf2 upregulation improves endothelial cell viability and promotes VEGF and endothelial nitric oxide synthase (eNOS) expression

**Figure 6:**
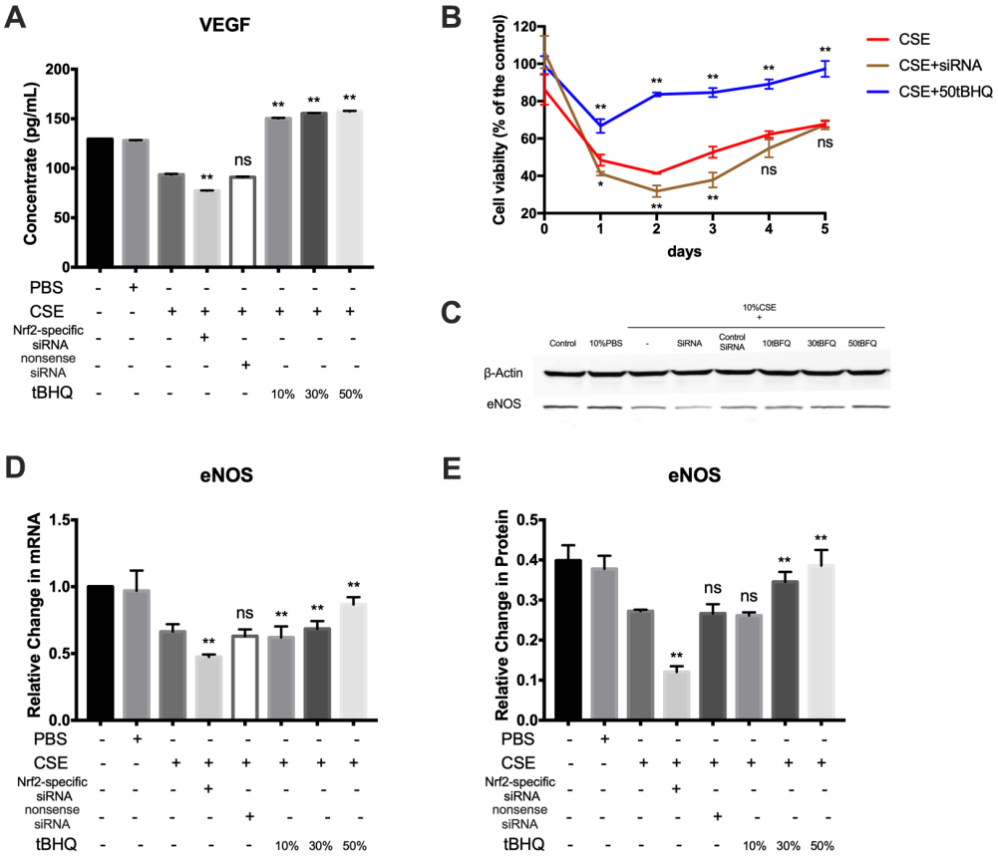
**Nrf2 upregulation improves endothelial cell viability and the expression of VEGF and eNOS.** (**A**) The expression of VEGF proteins was measured by ELISA. (**B**) Effect of Nrf2 on cell growth of HAECs treated by CSE was analyzed by measuring cell viability. (**C**–**E**) The mRNA and protein level of eNOS were detected by RT-PCR and Western blot. *p < 0.05, **p < 0.01, *ns*, not significant, compared with CSE group. The data are represented as mean ± SD (*n* = 3).

VEGF is a mitogenic factor that selectively drives endothelial cell growth, vascular permeability, and angiogenesis [23]. In this study, analysis of VEGF protein expression by ELISA revealed lower levels of VEGF in the CSE group relative to the control and PBS groups; further, Nrf2 could positively regulate the expression of VEGF (Figure 6A). To further demonstrate the cytotoxic effect of CSE against HAECs, the effect of CSE on the viability of HAECs at 1–5 days after treatment was analyzed using the CCK8 assay. Cell viability in the CSE group was decreased relative to controls; further, cell viability was improved in the tBHQ-treated groups but was decreased in the Nrf2-specific siRNA group relative to the CSE group (Figure 6B). eNOS is an essential regulator of endothelial function, serving as a key regulator of homeostasis and vascular tone by catalyzing NO production [24]. As shown in Figure 6C–6E, CSE treatment effectively downregulated eNOS at both the mRNA and protein levels, whereas Nrf2 could increase the expression level of eNOS. These results suggest that CSE induces a marked drop in cell viability and the expression of VEGF and eNOS in HAECs, and that these outcomes may be prevented by activating the Nrf2-related pathway.

### *In vivo* analysis

### Melatonin reduces rat carotid artery intimal hyperplasia following balloon injury with cigarette smoke exposure

**Figure 7:**
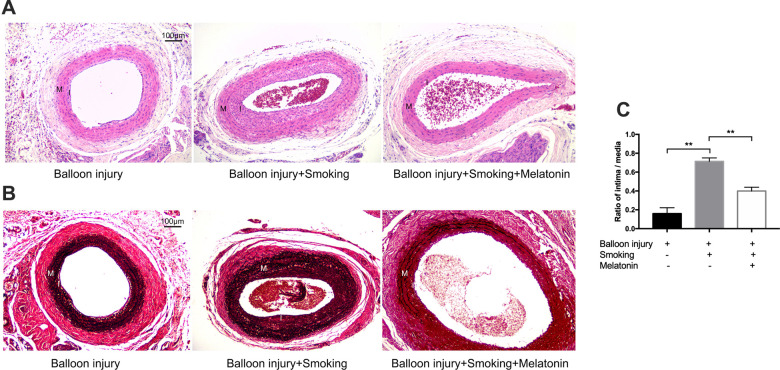
**Melatonin reduced intimal hyperplasia of rat carotid artery after balloon injury with cigarette smoking.** (**A**, **B**) H&E and VVG stained sections of carotid arteries of each group. Intima and media were labeled with "I" and "M" respectively. (**C**) Quantitative graphs of Intima to Media (I/M) ratio and intimal area. **p < 0.01. The data are represented as mean ± SD (*n* = 3).

To dissect the role of cigarette smoking during the progression of arterial remodeling and endothelial cell proliferation, we generated a rat carotid balloon injury model with cigarette smoke exposure. Further, the function of melatonin in cigarette-induced injury was investigated. H&E and Verhoeff von Gieson (VVG) staining were used to evaluate the histopathological changes in the carotid artery after smoke exposure for 4 weeks. H&E staining revealed variable levels of intimal hyperplasia in these treatment groups (Figure 7A). Figure 7B shows the VVG staining for elastin fibers. Staining of the external and internal elastic lamina (EEL and IEL) using VVG revealed the neointimal thickness. Cigarette smoke exposure increased the carotid artery neointima in the balloon injury model, and melatonin alleviated the degree of neointima.

The intimal area to medial area (I/M) ratio was employed as a means of quantifying the extent of luminal stenosis. This ratio was markedly decreased in the melatonin-treated group relative to that in the smoking group (Figure 7C). The results showed that balloon injury-induced stenosis of carotid arteries was largely aggravated by cigarette smoke exposure, but melatonin retarded this process.

### Melatonin activates the Nrf2/HO-1 pathway and decreases ROS levels

**Figure 8:**
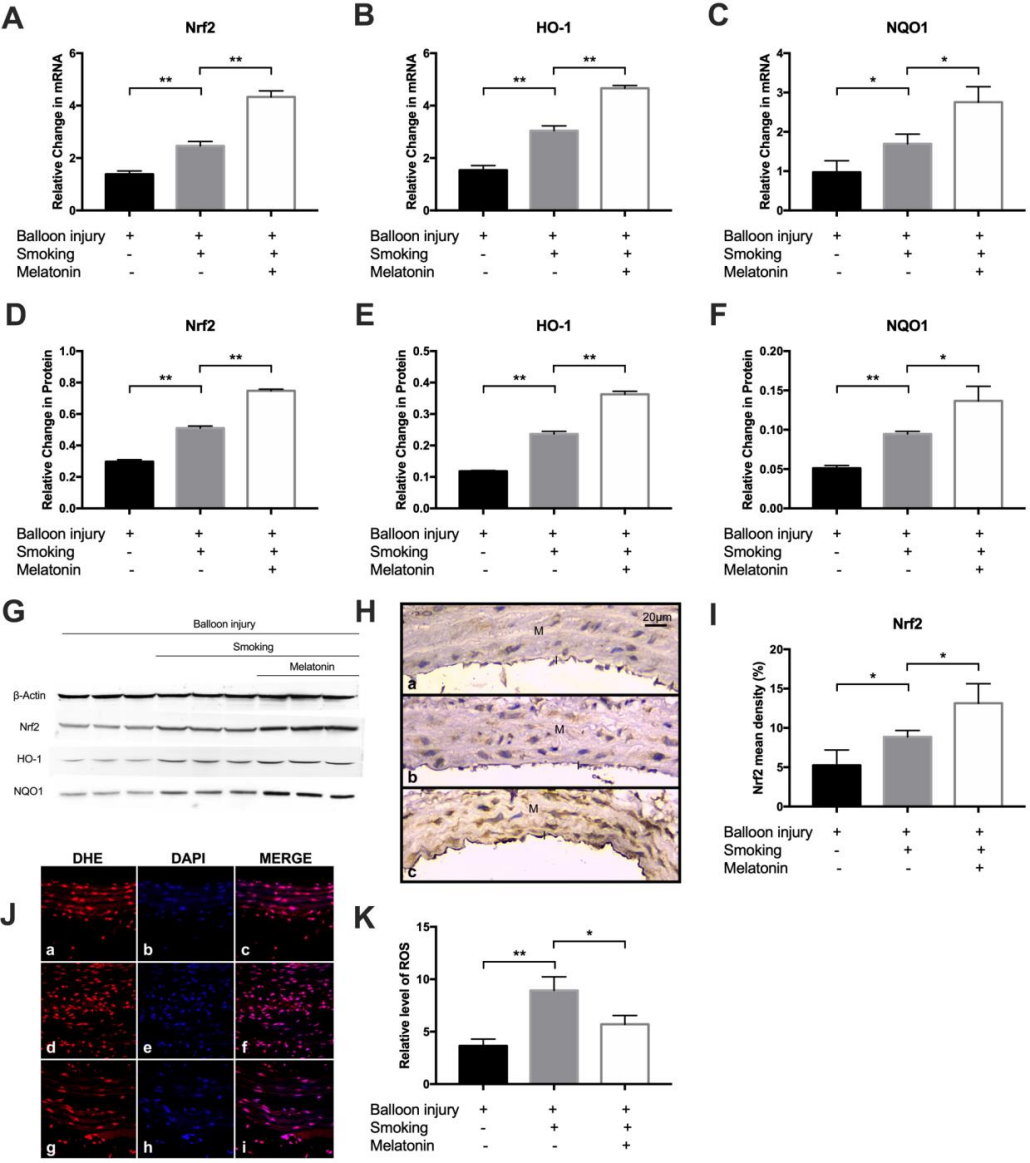
**Melatonin promoted expression of Nrf2/HO-1 pathway and decreased ROS level *in vivo*.** (**A**–**C**) The mRNA level of Nrf2, HO-1 and NQO1 was detected by RT-PCR. (**D**–**G**) The protein level of Nrf2, HO-1 and NQO1 were detected by Western blot. (**H**, **I**) Nrf-2 expression were detected by immunohistochemical staining (magnification ×400). Intima and media were labeled with "I" and "M" respectively. Nrf-2 mean density was analyzed in each group (a, control group; b, smoking group; c, melatonin group). (**J**, **K**) Representative images of carotid arteries stained with DHE (red), DAPI (blue) and merge images in (**a**–**c**) control, (**d**–**f**) smoking, and (**g**–**i**) melatonin groups. The fluorescent intensities of ROS were quantified. *p < 0.05, **p < 0.01. The data are represented as mean ± SD (*n* = 3).

We then investigated the impact of melatonin on the induction of Nrf2 expression in response to cigarette smoke *in vivo*. The mRNA and protein levels of Nrf2/HO-1 pathway components in the carotid arteries of the three groups was evaluated via RT-PCR and western blotting, respectively, following cigarette smoke exposure for 4 weeks. A marked rise in Nrf2, HO-1, and NQO1 expression was observed in the smoking group relative to controls; melatonin further promoted Nrf2/HO-1 pathway component expression (Figure 8A–8G). Immunohistochemical staining for Nrf2 showed that Nrf2 immunopositivity was localized to endothelial cells and vascular smooth muscle cells in the intima (cytoplasmic). The results showed a significantly increased level of Nrf2 in the melatonin group than in the smoking group (Figure 8H, 8I). Next, we explored carotid artery ROS levels using the fluorescent probe, DHE. Notably, controls exhibited low basal ROS levels. However, ROS generation was increased markedly in the smoking group, and this effect was reduced upon treatment with melatonin (Figure 8J, 8K). These data show that melatonin treatment inhibits smoking-induced ROS generation.

### Melatonin inhibits the expression of the NLRP3 inflammasome

**Figure 9:**
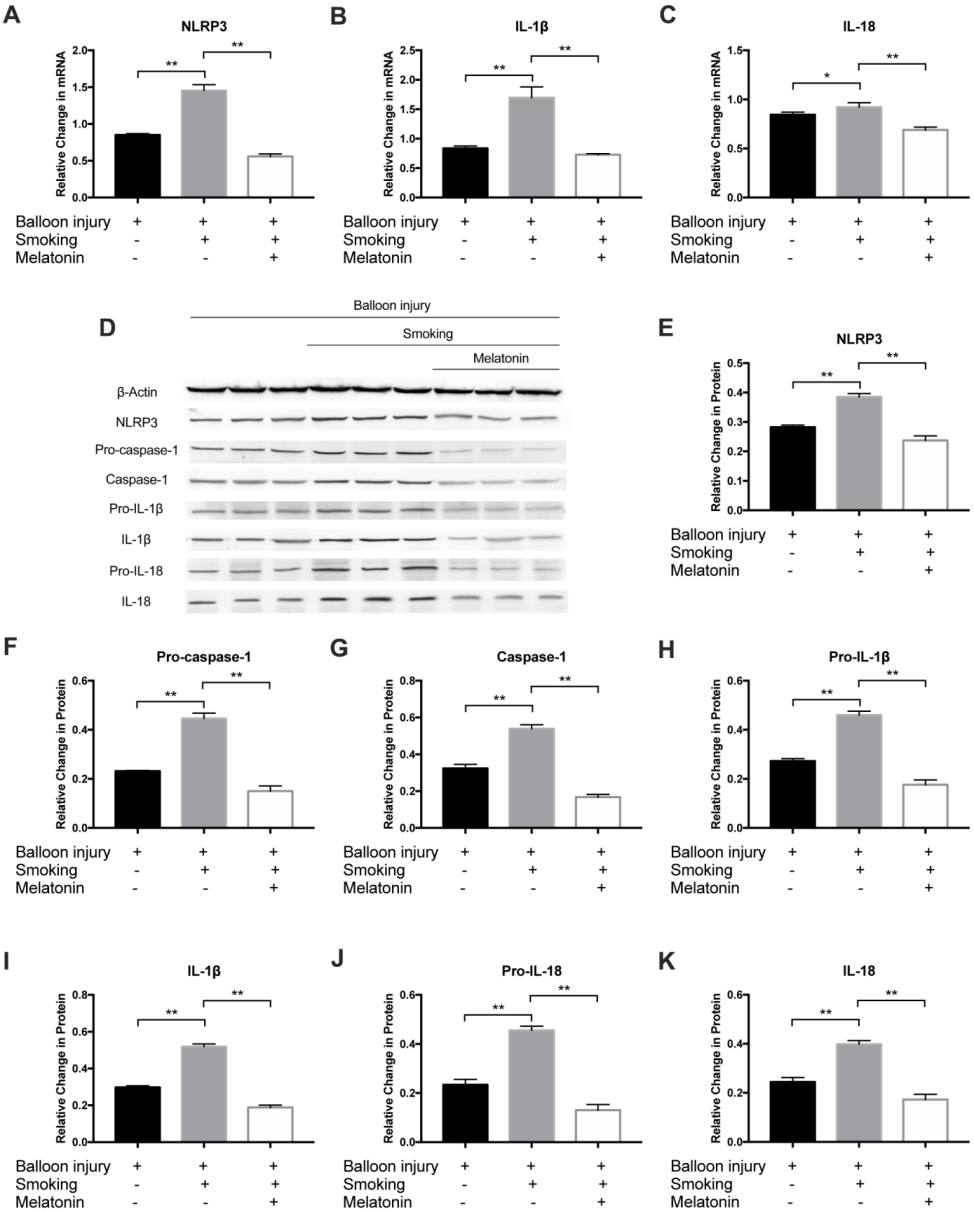
**Melatonin inhibited expression of NLRP3 inflammasome *in vivo*.** (**A**–**C**) The mRNA level of NLRP3, IL-1β and IL-18 was detected by RT-PCR. (**D**–**K**) NLRP3, Pro-Caspase-1, Caspase-1, Pro-IL-1β, IL-1β, Pro-IL-18 and IL-18 were detected by Western blot. *p < 0.05, **p < 0.01. The data are represented as mean ± SD (*n* = 3).

Moreover, to further validate the effect of melatonin on smoking-induced inflammatory cytokines, the NLRP3 inflammasome and downstream inflammatory factors were detected after cigarette smoke exposure for 4 weeks. Relative to the smoking group, NLRP3, IL-1β, and IL-18 mRNA levels were markedly decreased in the melatonin group (Figure 9A–9C). Western blot analysis revealed decreased NLRP3, pro-caspase-1, caspase-1, pro-IL-1β, IL-1β, pro-IL-18, and IL-18 protein levels in the melatonin group (Figure 9D–9K). As such, exposure to cigarette smoke can remarkably increase inflammation within carotid arteries, while melatonin protects against such injury via suppressing NLRP3 inflammasome activation.

## DISCUSSION

Smoking is a key risk factor associated with atherosclerotic progression and cardiovascular disease onset [25]. Recent work has shown that the underlying mechanisms include oxidative stress, vascular inflammation, and vascular dysfunction [26–28]. However, cigarette smoking is still probably the least understood among the risk factors for cardiovascular diseases. This is because cigarette smoke contains >7000 constituents and their contribution to tobacco dependence has not been studied thoroughly [29]. However, most studies use nicotine alone because it is the main constituent in tobacco. In this study, experimental models exposed to CSE and cigarette smoke were used to address the gap in smoking-related mechanisms between the real world and preclinical studies.

Prior studies have noted the importance of NLRP3 inflammasomes as regulators of many inflammatory diseases such as liver injury [30], autoimmune disease [31], and neurodegenerative disease [32]. Our *in vivo* study showed that NLRP3 inflammasome-mediated endothelial cell pyroptosis may be involved in atherosclerosis progression. A previous study found that cholesterol crystals accumulated in the intima in the early stage of atherosclerosis can activate the inflammatory response of macrophages and smooth muscle cells through NLRP3 inflammasomes, and eventually lead to the formation of unstable plaques [33]. While the majority of atherosclerosis research has assessed inflammasome activation within macrophages and monocytes, endothelial cell activation of this inflammasome in response to hyperglycemia was recently shown to be important as a regulator of the initiation of endothelial dysfunction [34]. It is known that NF-κB signaling is essential for the activation of the NLRP3 inflammasome [35]. Several reports have shown that ROS is a key regulator of such inflammasome activation [36, 37]. In accordance with these results, our previous studies have also demonstrated that cigarette smoking drives endothelial cell pyroptotic death via the ROS/NLRP3 axis [38]. Therefore, it is particularly important to find a suitable ROS scavenger to alleviate NLRP3 the inflammasome-associated vascular damage induced by smoking.

The Nrf2/HO-1 signaling pathway serves as a central regulator of antioxidant activity [9], and this pathway has also been increasingly shown to interact with the inflammasome pathways at multiple points. A recent study revealed that in cerebral ischemia reperfusion injury, Nrf2 suppresses the activation of the NLRP3 inflammasome by controlling the Trx1/TXNIP complex [39]. In addition, Chen et al. reported that suppressing Nrf2/HO-1 signaling in osteoarthritis enhances NLRP3 inflammasome signaling [40]. However, only a few studies have evaluated the interaction between Nrf2 and the NLRP3 inflammasome in smoking-induced vascular injury. Herein we saw that Nrf2/HO-1 pathway was stimulated by CSE treatment. We further observed that CSE-induced activation of ROS and the NLRP3 pathway were enhanced by Nrf2 siRNA, and inhibited by a Nrf2 inducer. Our study also revealed that HAECs displayed decreased functionality including inhibited cell viability as well as reduced secretion of VEGF and eNOS in the presence of CSE, whereas Nrf2 activation alleviated these changes. VEGF plays an important role in mediating angiogenesis and endothelial cell tube formation. VEGF is capable of stimulating the proliferation of endothelial cells at injured sites, thereby precluding excessive vascular smooth muscle cell proliferation and consequent intimal thickening and stenosis [41]. eNOS, mainly expressed by vascular endothelium, is a key enzyme in producing nitric oxide, which can regulate vascular tone as well as endothelial permeability [42]. Therefore, we speculated that the upregulation of the Nrf2 pathway can decrease CSE-mediated pyroptosis through the ROS/NLRP3 axis and may alleviate endothelial dysfunction. Nrf2 pathway targeting may thus be a potential strategy for alleviating the atherosclerotic lesions induced by smoking.

Recent studies have revealed that melatonin be beneficial in cardiovascular diseases, such as myocardial ischemia-reperfusion injury [43], hypertension [44], and atherosclerosis [45]. However, clinical studies on the cardioprotective effect of melatonin have shown different results. Two randomized placebo-controlled trials have confirmed the protective effects of melatonin during coronary artery bypass surgery [46] and percutaneous coronary intervention [47]. However, Ekeloef et al. demonstrated that compared with the placebo, in ST elevation myocardial infarction (STEMI) patients, melatonin failed to improve myocardial salvage indices following primary percutaneous coronary intervention [48]. Another study also showed that in a nonrestricted STEMI population, melatonin treatment was not associated with a reduction in infarct size and had an unfavorable effect on the ventricular volumes and evolution of the LVEF [49]. Therefore, more experiments are needed to additionally evaluate the intrinsic molecular mechanism underlying the activity of melatonin.

Melatonin exhibits antioxidant properties and is a potential modulator of Nrf2 [50]. Prior work has shown that Nrf2 signaling may mediate some of the therapeutic efficacy of melatonin in different diseases such as oxidative stress damage [51], ischemia and reperfusion injury [52], and neurotoxicity [53]. However, the influence of melatonin on smoking-induced vascular injury and atherosclerosis is not well reported. Our previous clinical study demonstrated that melatonin significantly increased the expression level of Nrf2 in smokers without causing any side effects [3]. Herein, we developed a balloon-induced carotid artery injury in smoking rats to mimic the tobacco toxicity to the vascular system. that the results showed that melatonin could reduce smoking-induced intimal hyperplasia in rats. Meanwhile, an enhanced Nrf2/HO-1 pathway and suppressed ROS/NLRP3 levels were observed with melatonin treatment, which may support the hypothesis that melatonin protects the vascular system from smoking-induced injury through the Nrf2/ROS/NLRP3 signaling pathway. Several reports have shown that melatonin activates the Nrf2 pathway through various other pathways. Shah et al. showed that Nrf2 activation and neuroprotection by melatonin are SIRT1-dependent in BV2 microglial cells [51]. It was also reported that melatonin upregulates the Nrf2/HO-1 pathway to protect mouse cortical astrocytes from hemin-induced toxicity by activating protein kinase C (PKC) α [54]. Further, Li et al. demonstrated that melatonin induced the phosphorylation of AMPK as well as the upregulation of Nrf2 to protect against chromium-induced cardiac injury [55]. However, further studies are needed to clarify the mechanistic basis for melatonin-mediated Nrf2 regulation in smoking-induced vascular injury.

**Figure 10:**
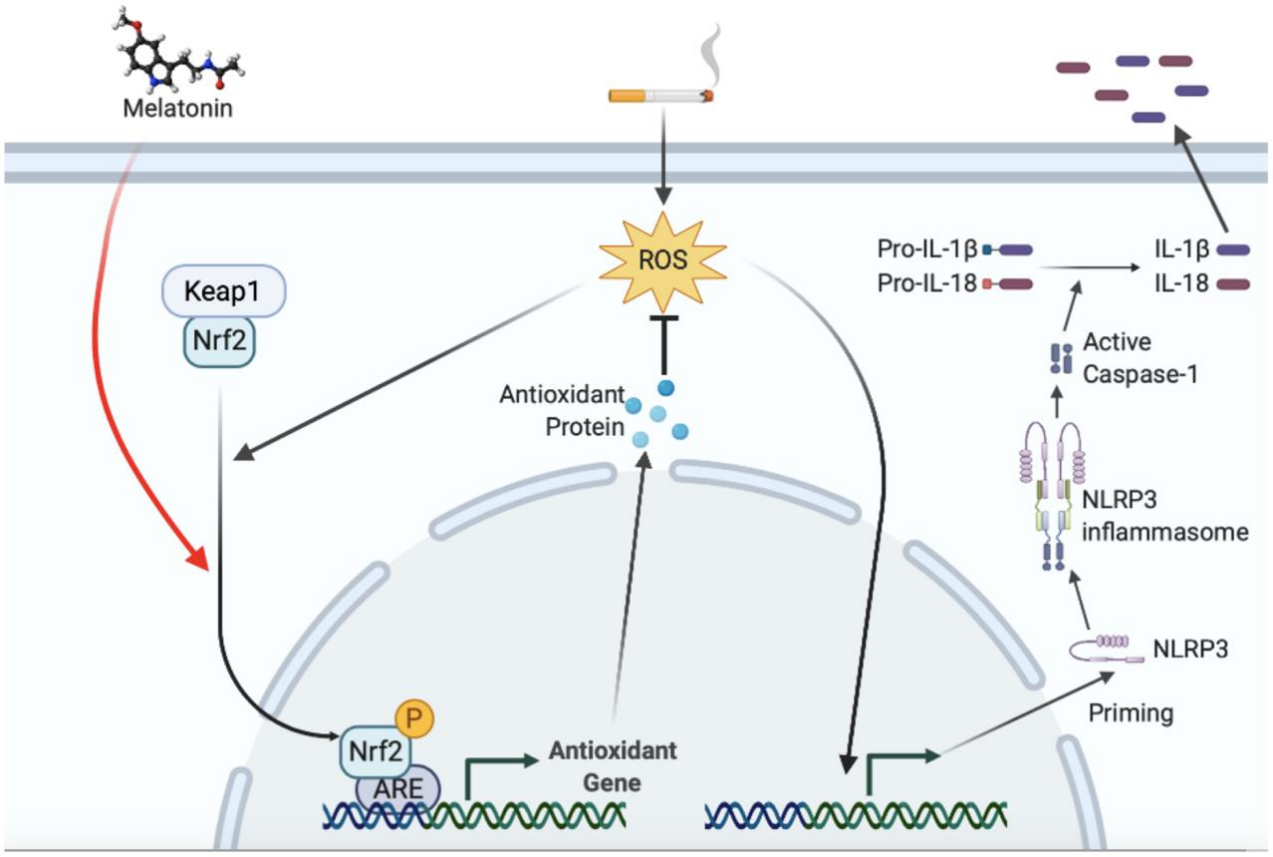
**Proposed model illustrating how melatonin protects smoking-related vascular inflammatory injury through Nrf2/ROS/NLRP3 axis.** ROS induced by smoking increased NLRP3 transcription and subsequent inflammasome activation, which promoted secretion of mature IL-1β and IL-18. ROS also stimulated the nuclear translocation of Nrf2 as a negative feedback. Melatonin induces the nuclear translocation of Nrf2, downstream expression, and elimination of intracellular ROS, resulting in a decrease in the transcription level of NLRP3 and downstream inflammasome activation.

In conclusion, smoking is thought to induce NLRP3-related pyroptosis in endothelial cells. Targeted regulation of the Nrf2 pathway may be a potential method to reduce the endothelial cell pyroptosis and vascular injury caused by smoking. A model was proposed to illustrate how melatonin protected smoking-related vascular injury and stenosis through the Nrf2/ROS/NLRP3 axis (Figure 10). Till now, there is no drug therapy for preventing smoking-induced atherosclerosis or restenosis after revascularization. Melatonin treatment might, therefore, be considered a potential therapeutic regimen with a high safety profile.

## MATERIALS AND METHODS

### Animal studies

All animal studies were performed in accordance with the Animal Management Rules of the Chinese Ministry of Health, and the Animal Care Committee of Peking Union Medical College approved all of these studies (No. XHDW-20150017). Male Sprague-Dawley (SD) rats (350-400 g) were obtained from and housed in the Laboratory Animal Center of Peking Union Medical College Hospital. Rats had free access to standard chow and tap water, and were housed in a climate-controlled facility (25–28° C, 12 h light/dark cycle).

Rats were randomized into a 3 groups (10 per group): control, smoking, and melatonin groups. All rats underwent balloon-induced carotid artery injury. Rats in the control group were exposed to air and administered DMSO, while rats in the smoking group were exposed to cigarettes smoke and administered DMSO, and rats in the melatonin group were exposed to cigarette smoke and administered melatonin (Sigma-Aldrich, MO, USA). Rats exposed to cigarette smoke were exposed to the equivalent smoke from 20 cigarettes per day (10 mg tar, 0.9 mg nicotine, 12 mg CO) over 120 minutes (from 9:00–10:00 and 15:00–16:00). Melatonin was prepared in normal saline containing 5% DMSO and diluted with saline prior to injection at 10 mg/kg/day (i.p.) [56]. Seven days after smoke exposure, the three groups were subjected to a previously detailed balloon-induced carotid artery injury [19]. At the 4th week following injury, animals were euthanized with pentobarbital sodium injection, and 2 cm carotid artery samples were separated and harvested. The samples were then fixed with 4% paraformaldehyde (PFA) 24 h or stored in liquid nitrogen.

### Cell lines and culture

HAECs were from the Institute of Basic Medical Sciences (Chinese Academy of Medical Sciences and Peking Union Medical College) and were cultured at 37° C in Endothelial Cell Medium (ECM, ScienCell Research Laboratories, CA, USA) containing 10% FBS, 1% endothelial cell growth factors, and penicillin/streptomycin in 5% CO_2_ and 95% air. Cigarette smoke extract (CSE) was generated as in prior reports using six filtered cigarettes (10 mg tar, 0.9 mg nicotine, 12 mg CO) [57]. HAECs were treated with Nrf2 siRNA or various concentrations of tertiary butylhydroquinone (tBHQ) before exposure to CSE. After 24 h of pre-treatment, media was replaced with fresh media supplemented with 10% CSE.

### Nrf2 siRNA interference

The pre-designed Nrf2 siRNA and the negative control were purchased from HippoBio (Zhejiang, China). Cells were grown to 80% confluence in 6-cm plates prior to washing with PBS and incubation with 2 mL of fresh basal medium. Subsequently, siRNA transfection was conducted using Lipofectamine RNAiMAX (Invitrogen, CA, USA) based on provided directions.

### Transmission electron microscopy (TEM) analysis

TEM can be employed to assess cell structures in pyroptosis. HAECs from different treatment groups were fixed with 2.5% glutaraldehyde in 0.1 M PBS overnight at 4° C, fixed with 1% OsO_4_ for 2 h, and rinsed twice with water for 15 min at room temperature. The samples were then dehydrated via ethanol gradient prior to epoxy resin embedding. Finally, the ultrathin sections (70 nm) were dual-stained using uranyl acetate and lead citrate and examined with a JEOL 1230 TEM (JEOL, Japan).

### qRT-PCR

TRIzol (Invitrogen) was used to extract total cell RNA, followed by quantification thereof with a NanoDrop 2000 instrument (Thermo Scientific, Bremen, Germany). cDNA was synthesized using SuperScript III RT (Invitrogen, CA, USA). Oligo(dT) was utilized during reverse transcription, and an Applied Biosystems StepOne Real-Time PCR instrument (Applied Biosystems Inc., CA, USA) was used for qRT-PCR with the SYBR qPCR Mix (Invitrogen) and the following forward (F) and reverse (R) primers: Nrf2, F 5′-TGCCCACATTCCCAAACAAG-3′ and 5′-GCTATCGAGTGACTGAGCCT-3′; HO-1, F 5′-CGCCTCCAGAGTTTCCGCCTCCAAC-3′, R 5′-ATAGACTGGGTTCTGCTTGTTTCGC-3′; NQO1, F 5′-CTTTCCGAAGCATTTCAGGGT CGTC-3′, R 5′-GCAGCTGGTTGCCAGGACGACCCTG-3′; NLRP3, F 5′-AAGGAAGTGGAC TGCGAGAA-3′ and R 5′-AACGTTCGTCCTTCCTTCCT-3′; IL-1β, F 5′-GGGATGATGACGACCTGCTA-3′ and R 5′-TGTCGTTGCTTGTCTCTCCT-3′; IL-18, F 5′-GCTGCCATGTCAGAAGAAGG-3′ and R 5′-GAACACAGGCGGGTTTCTTT-3′; eNOS, F 5′-GCTGGCATGGGCAACTTGAAGAGTG-3′, R 5′-CTGTCCTGCAGTCCCGAGCATCAAA-3′; β-actin, F 5′-CCAGCCTTCCTTCTTGGGTA-3′ and R 5′-CAATGCCTGGGTACA TGGTG-3′. β-actin served as an endogenous normalization control.

### Western blotting analysis

To assess Nrf2 translocation to the nucleus, a cytoplasmic extraction buffer with protease inhibitors (MDL biotech, Beijing, China) was used to lyse cells, and the lysate was centrifuged, and supernatants containing the cytosolic fraction were collected, while pelleted nuclei were rinsed thrice with PBS and lysed using RIPA buffer containing protease inhibitor cocktail (MDL biotech, Beijing, China). Total protein was extracted using lysis buffer (2% SDS, 10% glycerol, 62.5 mmol/L Tris-HCl; pH 6.8). After centrifugation at 4° C at 12,000 × *g* for 15 min, the supernatants were collected and protein levels therein were evaluated via BCA assay (MDL biotech). Protein samples (20 μg) were separated by 15% SDS-PAGe and transferred to PVDF membranes that were probed overnight at 4° C in primary antibodies directed against Nrf2 (1:1000, ab89443), HO-1 (1:2000, ab13243), NQO1 (1:1000, ab28947), NLRP3 (1:1000, ab232401), ASC (1:2000, ab180799), pro-caspase-1 (1:1000, ab179515), caspase-1 (1:1000, ab238979), pro-IL-1β (1:1000, Cat. No.: ab200284), IL-1β (1:1000, Cat. No.: ab2105), pro-IL-18 (1:3000, LSBIO, WA, USA, Cat. No.: LS-C179849), IL-18 (1:3000, ab191860), and GSDMD (1:1000, Cat. No.: ab219800). All were from Abcam (MA, USA) unless otherwise noted. Anti-β-actin (1:5000, MDL biotech, Beijing, China) was used as the loading control in each experiment. After three wash steps utilizing PBS, HRP-linked goat anti-mouse/anti-rabbit IgG (1:4000, MDL Biotech) was used to probe blots at room temperature for 1 h, after which enhanced chemiluminescence was utilized to detect protein bands.

### Detection of ROS production

ROS generation was assessed with DHE (dihydroethidium), which is a fluorescent probe sensitive to ROS. Cells were seeded in 6-well plates (6 × 10^4^ per well) and were treated as above for 24 h, followed by washing and incubation for 45 min with 10 μM DHE at 37° C. After oxidation, DHE can diffuse into cells wherein it can be detected via flow cytometry. Similarly, carotid artery sections were treated with 10 μM DHE in PBS for 45 min at 37° C, followed by three PBS washes. DAPI was used to stain nuclei, after which ROS levels were assessed by fluorescence microscopy. Images were captured via Leica DM3000 microscope (Leica Microsystems GmbH, Wetzlar, Germany). Three high-power fields (HPFs) (400× for immunofluorescence studies) randomly selected in a blinded manner were analyzed in each section. The mean intensity of red fluorescent areas per HPF was established for each animal.

### ELISA

HAECs were collected after treatment with CSE. Supernatant VEGF levels were assessed via ELISA kit (Cloud-Clone Corp., Hubei, China) based on provided directions.

### Cell viability assay

A CCK-8 assay was employed to quantify cellular survival. Cells were added to 96-well plates (2000/well) for 24 h, after which they were treated for 48 h with media containing CSE. Then, 10% CCK8 solution (Fluorescence Biotechnology Co. Ltd, Beijing, China) was added per well, and plates were incubated for 1 h at 37° C. Absorbance at 450 nm was then measured, and viability was then calculated as a metric for proliferation as follows: Cell viability = [A (CSE) – A (blank)] / [A (PBS) - A (blank)].

### Histopathological staining

After collection, tissue samples were subjected to standard treatment with an ethanol gradient, xylene, and paraffin-embedding. Following embedding, these tissues were cut into serial 4 μm sections and mounted. Hematoxylin-eosin (H&E) and Verhoeff von Gieson (VVG) staining were performed, respectively. Intimal thickening was assessed by measuring areas circumscribed by EEL, IEL, and the lumen area with Image-Pro Plus 6.0. The intima to media area (I/M) ratio was determined as follows: I/M ratio (%) = [IEL area − lumen area]/[EEL area − IEL area].

### Immunohistochemistry

For immunohistochemistry (IHC) analysis, paraffinized sections were mounted on glass slides and then deparaffinized. Following a 30 min blocking step using 5% goat serum in PBS at room temperature, blots were probed with primary anti-Nrf2 (1:100, Abcam, MA, USA, Cat. No. ab602352) overnight at 4° C. Next, HRP-linked secondary goat anti-rabbit IgG (1:200, MDL Biotech, Beijing, China) was used to probe sections, after which the 3,3′-diaminobenzidine tetrachloride chromogen was applied as a substrate at room temperature. Three fields of each sections were randomly selected for evaluation in a blinded manner. The expression levels of the factors were quantified with integrated optical density values from Image-Pro Plus 6.0.

### Statistical analysis

Continuous variables were given as means ± SD and compared by one-way ANOVAs and Tukey’s post hoc test. SPSS (version 19.0, SPSS software, Munich, Germany) was utilized for all testing. P < 0.05 was the significance threshold.

## References

[1] Chen Z, Peto R, Zhou M, Iona A, Smith M, Yang L, Guo Y, Chen Y, Bian Z, Lancaster G, Sherliker P, Pang S, Wang H, et al, and China Kadoorie Biobank (CKB) collaborative group. Contrasting male and female trends in tobacco-attributed mortality in China: evidence from successive nationwide prospective cohort studies. Lancet. 2015; 386:1447–56. 10.1016/S0140-6736(15)00340-2 26466050 PMC4691901

[2] Kianoush S, Yakoob MY, Al-Rifai M, DeFilippis AP, Bittencourt MS, Duncan BB, Bensenor IM, Bhatnagar A, Lotufo PA, Blaha MJ. Associations of cigarette smoking with subclinical inflammation and atherosclerosis: ELSA-Brasil (the Brazilian longitudinal study of adult health). J Am Heart Assoc. 2017; 6:e005088. 10.1161/JAHA.116.005088 28647689 PMC5669156

[3] Wang Z, Ni L, Wang J, Lu C, Ren M, Han W, Liu C. The protective effect of melatonin on smoke-induced vascular injury in rats and humans: a randomized controlled trial. J Pineal Res. 2016; 60:217–27. 10.1111/jpi.12305 26681403

[4] Yakala GK, Cabrera-Fuentes HA, Crespo-Avilan GE, Rattanasopa C, Burlacu A, George BL, Anand K, Mayan DC, Corlianò M, Hernández-Reséndiz S, Wu Z, Schwerk AM, Tan AL, et al. FURIN inhibition reduces vascular remodeling and atherosclerotic lesion progression in mice. Arterioscler Thromb Vasc Biol. 2019; 39:387–401. 10.1161/ATVBAHA.118.311903 30651003 PMC6393193

[5] Xu YJ, Zheng L, Hu YW, Wang Q. Pyroptosis and its relationship to atherosclerosis. Clin Chim Acta. 2018; 476:28–37. 10.1016/j.cca.2017.11.005 29129476

[6] Shi J, Gao W, Shao F. Pyroptosis: gasdermin-mediated programmed necrotic cell death. Trends Biochem Sci. 2017; 42:245–54. 10.1016/j.tibs.2016.10.004 27932073

[7] Cui M, Cui R, Liu K, Dong JY, Imano H, Hayama-Terada M, Muraki I, Kiyama M, Okada T, Kitamura A, Umesawa M, Yamagishi K, Ohira T, Iso H, and CIRCS investigators. Associations of tobacco smoking with impaired endothelial function: the circulatory risk in communities study (CIRCS). J Atheroscler Thromb. 2018; 25:836–45. 10.5551/jat.42150 29415955 PMC6143782

[8] Wu X, Zhang H, Qi W, Zhang Y, Li J, Li Z, Lin Y, Bai X, Liu X, Chen X, Yang H, Xu C, Zhang Y, Yang B. Nicotine promotes atherosclerosis via ROS-NLRP3-mediated endothelial cell pyroptosis. Cell Death Dis. 2018; 9:171. 10.1038/s41419-017-0257-3 29416034 PMC5833729

[9] Loboda A, Damulewicz M, Pyza E, Jozkowicz A, Dulak J. Role of Nrf2/HO-1 system in development, oxidative stress response and diseases: an evolutionarily conserved mechanism. Cell Mol Life Sci. 2016; 73:3221–47. 10.1007/s00018-016-2223-0 27100828 PMC4967105

[10] Pogu J, Tzima S, Kollias G, Anegon I, Blancou P, Simon T. Genetic restoration of heme oxygenase-1 expression protects from type 1 diabetes in NOD mice. Int J Mol Sci. 2019; 20:1676. 10.3390/ijms20071676 30987262 PMC6480274

[11] Xu G, Zhao X, Fu J, Wang X. Resveratrol increase myocardial Nrf2 expression in type 2 diabetic rats and alleviate myocardial ischemia/reperfusion injury (MIRI). Ann Palliat Med. 2019; 8:565–75. 10.21037/apm.2019.11.25 31865720

[12] Ling Y, Li ZZ, Zhang JF, Zheng XW, Lei ZQ, Chen RY, Feng JH. MicroRNA-494 inhibition alleviates acute lung injury through Nrf2 signaling pathway via NQO1 in sepsis-associated acute respiratory distress syndrome. Life Sci. 2018; 210:1–8. 10.1016/j.lfs.2018.08.037 30121199 PMC9673760

[13] Deshmukh P, Unni S, Krishnappa G, Padmanabhan B. The Keap1-Nrf2 pathway: promising therapeutic target to counteract ROS-mediated damage in cancers and neurodegenerative diseases. Biophys Rev. 2017; 9:41–56. 10.1007/s12551-016-0244-4 28510041 PMC5425799

[14] Cui W, Zhang Z, Zhang P, Qu J, Zheng C, Mo X, Zhou W, Xu L, Yao H, Gao J. Nrf2 attenuates inflammatory response in COPD/emphysema: crosstalk with Wnt3a/β-catenin and AMPK pathways. J Cell Mol Med. 2018; 22:3514–25. 10.1111/jcmm.13628 29659176 PMC6010849

[15] Sakurai H, Morishima Y, Ishii Y, Yoshida K, Nakajima M, Tsunoda Y, Hayashi SY, Kiwamoto T, Matsuno Y, Kawaguchi M, Yamamoto M, Hizawa N. Sulforaphane ameliorates steroid insensitivity through an Nrf2-dependent pathway in cigarette smoke-exposed asthmatic mice. Free Radic Biol Med. 2018; 129:473–85. 10.1016/j.freeradbiomed.2018.10.400 30312763

[16] Piotrowicz K, Klich-Rączka A, Pac A, Zdzienicka A, Grodzicki T. The diurnal profile of melatonin during delirium in elderly patients—preliminary results. Exp Gerontol. 2015; 72:45–49. 10.1016/j.exger.2015.09.007 26368540

[17] Reiter RJ, Mayo JC, Tan DX, Sainz RM, Alatorre-Jimenez M, Qin L. Melatonin as an antioxidant: under promises but over delivers. J Pineal Res. 2016; 61:253–78. 10.1111/jpi.12360 27500468

[18] Andersen LP, Gögenur I, Rosenberg J, Reiter RJ. The safety of melatonin in humans. Clin Drug Investig. 2016; 36:169–75. 10.1007/s40261-015-0368-5 26692007

[19] Yang GH, Li YC, Wang ZQ, Liu B, Ye W, Ni L, Zeng R, Miao SY, Wang LF, Liu CW. Protective effect of melatonin on cigarette smoke-induced restenosis in rat carotid arteries after balloon injury. J Pineal Res. 2014; 57:451–58. 10.1111/jpi.12185 25251422

[20] Qiao L, Wu X, Zhang J, Liu L, Sui X, Zhang R, Liu W, Shen F, Sun Y, Xi X. α-NETA induces pyroptosis of epithelial ovarian cancer cells through the GSDMD/caspase-4 pathway. FASEB J. 2019; 33:12760–67. 10.1096/fj.201900483RR 31480859

[21] Wang S, Yuan YH, Chen NH, Wang HB. The mechanisms of NLRP3 inflammasome/pyroptosis activation and their role in Parkinson’s disease. Int Immunopharmacol. 2019; 67:458–64. 10.1016/j.intimp.2018.12.019 30594776

[22] Lin XX, Yang XF, Jiang JX, Zhang SJ, Guan Y, Liu YN, Sun YH, Xie QM. Cigarette smoke extract-induced BEAS-2B cell apoptosis and anti-oxidative Nrf-2 up-regulation are mediated by ROS-stimulated p38 activation. Toxicol Mech Methods. 2014; 24:575–83. 10.3109/15376516.2014.956909 25134437

[23] Wang S, Lu J, You Q, Huang H, Chen Y, Liu K. The mTOR/AP-1/VEGF signaling pathway regulates vascular endothelial cell growth. Oncotarget. 2016; 7:53269–76. 10.18632/oncotarget.10756 27458160 PMC5288184

[24] Siragusa M, Fleming I. The eNOS signalosome and its link to endothelial dysfunction. Pflugers Arch. 2016; 468:1125–37. 10.1007/s00424-016-1839-0 27184745

[25] Siasos G, Tsigkou V, Kokkou E, Oikonomou E, Vavuranakis M, Vlachopoulos C, Verveniotis A, Limperi M, Genimata V, Papavassiliou AG, Stefanadis C, Tousoulis D. Smoking and atherosclerosis: mechanisms of disease and new therapeutic approaches. Curr Med Chem. 2014; 21:3936–48. 10.2174/092986732134141015161539 25174928

[26] Messner B, Bernhard D. Smoking and cardiovascular disease: mechanisms of endothelial dysfunction and early atherogenesis. Arterioscler Thromb Vasc Biol. 2014; 34:509–15. 10.1161/ATVBAHA.113.300156 24554606

[27] Adams T, Wan E, Wei Y, Wahab R, Castagna F, Wang G, Emin M, Russo C, Homma S, Le Jemtel TH, Jelic S. Secondhand smoking is associated with vascular inflammation. Chest. 2015; 148:112–19. 10.1378/chest.14-2045 25742439 PMC4493867

[28] Dikalov S, Itani H, Richmond B, Vergeade A, Rahman SM, Boutaud O, Blackwell T, Massion PP, Harrison DG, Dikalova A. Tobacco smoking induces cardiovascular mitochondrial oxidative stress, promotes endothelial dysfunction, and enhances hypertension. Am J Physiol Heart Circ Physiol. 2019; 316:H639–46. 10.1152/ajpheart.00595.2018 30608177 PMC6459311

[29] U.S.DHSS. How Tobacco Smoke Causes Disease The Biology and Behavioral Basis for Smoking-Attributable Disease A Report of the Surgeon General. Public Health. 2010. 21452462

[30] Wree A, McGeough MD, Inzaugarat ME, Eguchi A, Schuster S, Johnson CD, Peña CA, Geisler LJ, Papouchado BG, Hoffman HM, Feldstein AE. NLRP3 inflammasome driven liver injury and fibrosis: roles of IL-17 and TNF in mice. Hepatology. 2018; 67:736–49. 10.1002/hep.29523 28902427 PMC5849484

[31] Shen HH, Yang YX, Meng X, Luo XY, Li XM, Shuai ZW, Ye DQ, Pan HF. NLRP3: A promising therapeutic target for autoimmune diseases. Autoimmun Rev. 2018; 17:694–702. 10.1016/j.autrev.2018.01.020 29729449

[32] Heneka MT, Kummer MP, Stutz A, Delekate A, Schwartz S, Vieira-Saecker A, Griep A, Axt D, Remus A, Tzeng TC, Gelpi E, Halle A, Korte M, et al. NLRP3 is activated in Alzheimer’s disease and contributes to pathology in APP/PS1 mice. Nature. 2013; 493:674–78. 10.1038/nature11729 23254930 PMC3812809

[33] Janoudi A, Shamoun FE, Kalavakunta JK, Abela GS. Cholesterol crystal induced arterial inflammation and destabilization of atherosclerotic plaque. Eur Heart J. 2016; 37:1959–67. 10.1093/eurheartj/ehv653 26705388

[34] Chen Y, Wang L, Pitzer AL, Li X, Li PL, Zhang Y. Contribution of redox-dependent activation of endothelial Nlrp3 inflammasomes to hyperglycemia-induced endothelial dysfunction. J Mol Med (Berl). 2016; 94:1335–47. 10.1007/s00109-016-1481-5 27783111 PMC5512566

[35] Bauernfeind FG, Horvath G, Stutz A, Alnemri ES, MacDonald K, Speert D, Fernandes-Alnemri T, Wu J, Monks BG, Fitzgerald KA, Hornung V, Latz E. Cutting edge: NF-kappaB activating pattern recognition and cytokine receptors license NLRP3 inflammasome activation by regulating NLRP3 expression. J Immunol. 2009; 183:787–91. 10.4049/jimmunol.0901363 19570822 PMC2824855

[36] Qiu Z, He Y, Ming H, Lei S, Leng Y, Xia ZY. Lipopolysaccharide (LPS) aggravates high glucose- and hypoxia/reoxygenation-induced injury through activating ROS-dependent NLRP3 inflammasome-mediated pyroptosis in H9C2 cardiomyocytes. J Diabetes Res. 2019; 2019:8151836. 10.1155/2019/8151836 30911553 PMC6398034

[37] Minutoli L, Puzzolo D, Rinaldi M, Irrera N, Marini H, Arcoraci V, Bitto A, Crea G, Pisani A, Squadrito F, Trichilo V, Bruschetta D, Micali A, Altavilla D. ROS-mediated NLRP3 inflammasome activation in brain, heart, kidney, and testis ischemia/reperfusion injury. Oxid Med Cell Longev. 2016; 2016:2183026. 10.1155/2016/2183026 27127546 PMC4835650

[38] Wang X, Bian Y, Zhang R, Liu X, Ni L, Ma B, Zeng R, Zhao Z, Song X, Liu C. Melatonin alleviates cigarette smoke-induced endothelial cell pyroptosis through inhibiting ROS/NLRP3 axis. Biochem Biophys Res Commun. 2019; 519:402–08. 10.1016/j.bbrc.2019.09.005 31521245

[39] Hou Y, Wang Y, He Q, Li L, Xie H, Zhao Y, Zhao J. Nrf2 inhibits NLRP3 inflammasome activation through regulating Trx1/TXNIP complex in cerebral ischemia reperfusion injury. Behav Brain Res. 2018; 336:32–39. 10.1016/j.bbr.2017.06.027 28851669

[40] Chen Z, Zhong H, Wei J, Lin S, Zong Z, Gong F, Huang X, Sun J, Li P, Lin H, Wei B, Chu J. Inhibition of Nrf2/HO-1 signaling leads to increased activation of the NLRP3 inflammasome in osteoarthritis. Arthritis Res Ther. 2019; 21:300. 10.1186/s13075-019-2085-6 31870428 PMC6929452

[41] Tian S, Cao D, Zou H, Bai F, Wang Z, Pan S, Feng M. Endothelial cell-targeted pVEGF165 polyplex plays a pivotal role in inhibiting intimal thickening after vascular injury. Int J Nanomedicine. 2015; 10:5751–68. 10.2147/IJN.S88109 26425083 PMC4583553

[42] Stamer WD, Lei Y, Boussommier-Calleja A, Overby DR, Ethier CR. eNOS, a pressure-dependent regulator of intraocular pressure. Invest Ophthalmol Vis Sci. 2011; 52:9438–44. 10.1167/iovs.11-7839 22039240 PMC3293415

[43] Feng J, Chen X, Liu R, Cao C, Zhang W, Zhao Y, Nie S. Melatonin protects against myocardial ischemia-reperfusion injury by elevating Sirtuin3 expression and manganese superoxide dismutase activity. Free Radic Res. 2018; 52:840–49. 10.1080/10715762.2018.1461215 30208798

[44] Prado NJ, Ferder L, Manucha W, Diez ER. Anti-inflammatory effects of melatonin in obesity and hypertension. Curr Hypertens Rep. 2018; 20:45. 10.1007/s11906-018-0842-6 29744660

[45] Ma S, Chen J, Feng J, Zhang R, Fan M, Han D, Li X, Li C, Ren J, Wang Y, Cao F. Melatonin ameliorates the progression of atherosclerosis via mitophagy activation and NLRP3 inflammasome inhibition. Oxid Med Cell Longev. 2018; 2018:9286458. 10.1155/2018/9286458 30254716 PMC6142770

[46] Dwaich KH, Al-Amran FG, Al-Sheibani BI, Al-Aubaidy HA. Melatonin effects on myocardial ischemia-reperfusion injury: impact on the outcome in patients undergoing coronary artery bypass grafting surgery. Int J Cardiol. 2016; 221:977–86. 10.1016/j.ijcard.2016.07.108 27441478

[47] Dominguez-Rodriguez A, Abreu-Gonzalez P, de la Torre-Hernandez JM, Consuegra-Sanchez L, Piccolo R, Gonzalez-Gonzalez J, Garcia-Camarero T, Del Mar Garcia-Saiz M, Aldea-Perona A, Reiter RJ, Caballero-Estevez N, de la Rosa A, Virgos-Aller T, et al, and MARIA Investigators. Usefulness of Early Treatment With Melatonin to Reduce Infarct Size in Patients With ST-Segment Elevation Myocardial Infarction Receiving Percutaneous Coronary Intervention (From the Melatonin Adjunct in the Acute Myocardial Infarction Treated With Angioplasty Trial). Am J Cardiol. 2017; 120:522–26. 10.1016/j.amjcard.2017.05.018 28645475

[48] Ekeloef S, Halladin N, Fonnes S, Jensen SE, Zaremba T, Rosenberg J, Jonsson G, Aarøe J, Gasbjerg LS, Rosenkilde MM, Gögenur I. Effect of Intracoronary and Intravenous Melatonin on Myocardial Salvage Index in Patients with ST-Elevation Myocardial Infarction: a Randomized Placebo Controlled Trial. J Cardiovasc Transl Res. 2017; 10:470–79. 10.1007/s12265-017-9768-7 29027116

[49] Dominguez-Rodriguez A, Abreu-Gonzalez P, de la Torre-Hernandez JM, Gonzalez-Gonzalez J, Garcia-Camarero T, Consuegra-Sanchez L, Garcia-Saiz MD, Aldea-Perona A, Virgos-Aller T, Azpeitia A, Reiter RJ, and MARIA Investigators. Effect of intravenous and intracoronary melatonin as an adjunct to primary percutaneous coronary intervention for acute ST-elevation myocardial infarction: results of the Melatonin Adjunct in the acute myocaRdial Infarction treated with Angioplasty trial. J Pineal Res. 2017; 62:e12374. 10.1111/jpi.12374 27736028

[50] Ahmadi Z, Ashrafizadeh M. Melatonin as a potential modulator of Nrf2. Fundam Clin Pharmacol. 2020; 34:11–19. 10.1111/fcp.12498 31283051

[51] Shah SA, Khan M, Jo MH, Jo MG, Amin FU, Kim MO. Melatonin stimulates the SIRT1/Nrf2 signaling pathway counteracting lipopolysaccharide (LPS)-induced oxidative stress to rescue postnatal rat brain. CNS Neurosci Ther. 2017; 23:33–44. 10.1111/cns.12588 27421686 PMC6492734

[52] Shi S, Lei S, Tang C, Wang K, Xia Z. Melatonin attenuates acute kidney ischemia/reperfusion injury in diabetic rats by activation of the SIRT1/Nrf2/HO-1 signaling pathway. Biosci Rep. 2019; 39:BSR20181614. 10.1042/BSR20181614 30578379 PMC6331666

[53] Sadek KM, Lebda MA, Abouzed TK. The possible neuroprotective effects of melatonin in aluminum chloride-induced neurotoxicity via antioxidant pathway and Nrf2 signaling apart from metal chelation. Environ Sci Pollut Res Int. 2019; 26:9174–83. 10.1007/s11356-019-04430-9 30719664

[54] Chen X, Xi Z, Liang H, Sun Y, Zhong Z, Wang B, Bian L, Sun Q. Melatonin prevents mice cortical astrocytes from hemin-induced toxicity through activating PKCα/Nrf2/HO-1 signaling *in vitro*. Front Neurosci. 2019; 13:760. 10.3389/fnins.2019.00760 31404262 PMC6669962

[55] Li J, Zheng X, Ma X, Xu X, Du Y, Lv Q, Li X, Wu Y, Sun H, Yu L, Zhang Z. Melatonin protects against chromium(VI)-induced cardiac injury via activating the AMPK/Nrf2 pathway. J Inorg Biochem. 2019; 197:110698. 10.1016/j.jinorgbio.2019.110698 31054488

[56] Li T, Ni L, Zhao Z, Liu X, Lai Z, Di X, Xie Z, Song X, Wang X, Zhang R, Liu C. Melatonin attenuates smoking-induced hyperglycemia via preserving insulin secretion and hepatic glycogen synthesis in rats. J Pineal Res. 2018; 64:e12475. 10.1111/jpi.12475 29437243 PMC5947659

[57] Li T, Song T, Ni L, Yang G, Song X, Wu L, Liu B, Liu C. The p-ERK-p-c-Jun-cyclinD1 pathway is involved in proliferation of smooth muscle cells after exposure to cigarette smoke extract. Biochem Biophys Res Commun. 2014; 453:316–20. 10.1016/j.bbrc.2014.09.062 25260414

